# Develop a Compact RNA Base Editor by Fusing ADAR with Engineered EcCas6e

**DOI:** 10.1002/advs.202206813

**Published:** 2023-04-25

**Authors:** Xing Wang, Renxia Zhang, Dong Yang, Guoling Li, Zhanqing Fan, Hongting Du, Zikang Wang, Yuanhua Liu, Jiajia Lin, Xiaoqing Wu, Linyu Shi, Hui Yang, Yingsi Zhou

**Affiliations:** ^1^ HuidaGene Therapeutics Co. Ltd. 6th Floor, Unit 3, Building 5, No. 160 Basheng Road, Pudong New Area Shanghai 200131 China; ^2^ Institute of Neuroscience State Key Laboratory of Neuroscience Key Laboratory of Primate Neurobiology CAS Center for Excellence in Brain Science and Intelligence Technology Chinese Academy of Science 320 Yueyang Road Shanghai 200031 China; ^3^ Department of Neurology First Affiliated Hospital Fujian Medical University No. 20 Chazhong Road Fuzhou 350005 China

**Keywords:** CRISPR, gene therapy, RNA base editing

## Abstract

Catalytically inactive CRISPR‐Cas13 (dCas13)‐based base editors can achieve the conversion of adenine‐to‐inosine (A‐to‐I) or cytidine‐to‐uridine (C‐to‐U) at the RNA level, however, the large size of dCas13 protein limits its in vivo applications. Here, a compact and efficient RNA base editor (ceRBE) is reported with high in vivo editing efficiency. The larger dCas13 protein is replaced with a 199‐amino acid EcCas6e protein, derived from the Class 1 CRISPR family involved in pre‐crRNA processing, and conducted optimization for toxicity and editing efficiency. The ceRBE efficiently achieves both A‐to‐I and C‐to‐U base editing with low transcriptome off‐target in HEK293T cells. The efficient repair of the DMD Q1392X mutation (68.3±10.1%) is also demonstrated in a humanized mouse model of Duchenne muscular dystrophy (DMD) after AAV delivery, achieving restoration of expression for gene products. The study supports that the compact and efficient ceRBE has great potential for treating genetic diseases.

## Graphical abstract

For all CRISPR/Cas systems, Cas6 is the smallest crRNA‐binding protein with only 199 amino acids. Similar to Cas13 protein, Cas6 can also be developed into an efficient and high‐fidelity RNA base editor. Through AAV delivery, Cas6 base editor, ceRBE, can successfully treat humanized mouse model of Duchenne muscular dystrophy (DMD).

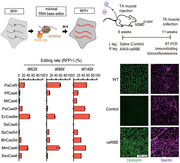

## 1 Introduction

RNA editing can modify gene products without causing permanent changes in the genome, and has great potential in gene therapy research. Among the numerous RNA base editing technologies, the base editor composed of catalytically inactive RNA‐targeting CRISPR‐Cas13 (dCas13) protein and a specific RNA deaminase has great advantages in efficiency and editing types.^[^ ^1^, ^2^, ^3^ ^]^ This strategy has achieved efficient adenine‐to‐inosine (A‐to‐I) or cytidine‐to‐uridine (C‐to‐U) base editing at specific sites in cells in vitro. However, the large size and low editing efficiency of dCas13‐based RNA base editors still limit its application in vivo therapy. Effectively delivering the RNA base editors to the specific tissues is crucial for its use as therapeutic drugs. Adeno‐associated virus (AAVs) is the most promising vehicles of gene therapy due to their simplicity, low genotoxicity, and long‐term gene expression.^[^ ^4^ ^]^ However, the size of most base editors, consisting of dCas13 protein binding to RNA deaminase, makes it difficult to package into AAV for efficient in vivo delivery.^[^ ^1^, ^2^, ^3^ ^]^ Therefore, a smaller RNA base editor with higher editing efficiency in vivo is needed.

The Cas13 protein is a crRNA‐mediated CRISPR nuclease targeting single‐stranded RNA for cleavage. Unlike Cas9, Cas13 processes its own pre‐crRNA into mature crRNAs without tracrRNA and RNase III. All Cas13 proteins carry a pre‐crRNA processing active site and a target RNA cleavage active site formed by two HEPN domains.^[^ ^5^, ^6^, ^7^ ^]^ Among all Cas13 proteins, Cas13X.1 is the smallest protein with 775 amino acids which is more suitable for AAV delivery.^[^ ^3^ ^]^ The HEPN‐inactive Cas13s have been engineered as programmable RNA‐binding modules for RNA base editing.^[^ ^1^, ^3^, ^8^ ^]^

The Cas6, Cas5d and Csf5 proteins are small endoribonucleases with around 200 amino acids that process pre‐crRNAs for Class 1 CRISPR‐Cas systems.^[^ ^9^ ^]^ In type I CRISPR‐Cas systems, the Cas6 is also a key component of Cascade complex.^[^ ^9^ ^]^ Cas6f (Csy4) was reported to have high‐affinity for its pre‐crRNA and mature crRNA with an equilibrium dissociation constant of 50 pM.^[^ ^10^ ^]^ By taking advantage of the hairpin specificity of the Cas6 proteins, Csy4 (Cas6f) and Cse3 (Cas6e) have been used for multiplexed crRNA release and RNA tracing.^[^ ^11^, ^12^ ^]^ Considering the small size of Cas6, we wonder whether Cas6 can be applied to RNA editing like Cas13.

Here, we describe a smaller RNA base editor with higher editing efficiency in vivo than the existing dCas13‐mediated RNA base editors – ceRBE. We replace the existing dCas13 protein with ten small pre‐crRNA processing proteins, and successfully achieve RNA base editing. The EcCas6e protein, with low off‐target and high editing efficiency, is selected to optimize for toxicity and editing efficiency. Compared with the RESCUE‐S editor with the same deaminase, the optimized ceRBE shows a higher A‐to‐I and C‐to‐U editing ratio in HEK293T cells (A‐to‐I, 36.7–60.3%; C‐to‐U, 35.7–54.3%) with lower transcriptome off‐target. Further, to determine the therapeutic potential of the editor, we package the editor into a single AAV and explore in vivo treatment of the DMD Q1392X mutation in the humanized mouse model of DMD. The proportion of dystrophin fibers recovers to 68.1±9.4% at three weeks after intramuscular injection. Overall, ceRBE, as a small and efficient RNA base editor, exhibits strong therapeutic potential for the genetic diseases.

## 2 Results

### 2.1 Small‐Sized DR‐Processing Proteins Fused to Adenine Deaminase Successfully Achieve RNA Base Editing in HEK293T Cells

**Figure 1:**
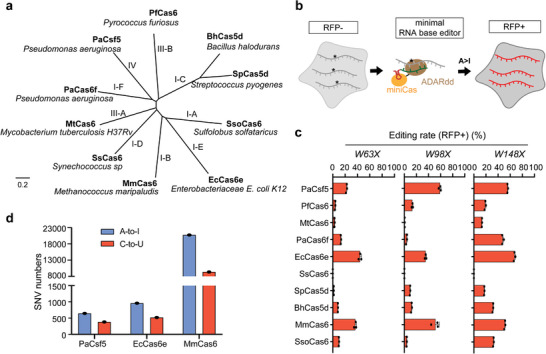
RNA base editing of small DR‐processing proteins fused to adenine deaminase in HEK293T cells. a) A phylogenetic tree of ten representative Cas proteins from each type or subtype of Class 1 CRISPR proteins, including SsoCas6, MmCas6, BhCas5d, SpCas5d, SsCas6, EcCas6e, PaCas6f, MtCas6, PfCas6, and PaCsf5 by multi‐sequence alignments using MAFFT^[^ ^30^ ^]^ and MEGA.^[^ ^31^ ^]^ The evolutionary distance scale of 0.2 is shown. b) Schematic of RNA base editing by the editor composed of small Cas protein and ADAR protein, the deaminase in the RESCUE‐S editor. Small Cas proteins can recognize the DR sequence of crRNA targeting a specific site. Only when the fusion‐expressed ADAR protein achieves A‐to‐I conversion and repairs the stop codon on mCherry mRNA, red fluorescent protein can be expressed. c) RNA editing efficiency of base editors with different small Cas proteins in HEK293T cells. Base editing efficiency is indicated by the positive proportion of red fluorescence in flow cytometry results. W63X, W98X and W148X indicate the mutations at different sites in mCherry coding sequence. Values represent mean ± SD; n = 3. d) Transcriptome‐wide off‐target sites numbers for PaCsf5, EcCas6e, and MmCas6 without targeting gRNA transfection experiments in HEK293T cells. n = 1. See Supporting Information Data 1 for detailed data.

To test this possibility, we selected ten representative Cas proteins from each type or subtype of Class 1 CRISPR proteins, including SsoCas6,^[^ ^13^ ^]^ MmCas6,^[^ ^14^ ^]^ BhCas5d,^[^ ^15^ ^]^ SpCas5d,^[^ ^16^ ^]^ SsCas6,^[^ ^17^ ^]^ EcCas6e,^[^ ^18^ ^]^ PaCas6f,^[^ ^19^ ^]^ MtCas6,^[^ ^20^ ^]^ PfCas6,^[^ ^21^ ^]^ and PaCsf5^[^ ^22^ ^]^ (**Figure** **1**a and Table S1, Supporting Information).

We generated mammalian codon‐optimized versions of ten Cas proteins, and fused it with the RNA deaminase, which is used in RESCUE‐S base editor (henceforth termed “ADAR^RESCUE‐S^”).^[^ ^2^ ^]^ We further added a nuclear localization signal (NLS) to both ends of the fusion proteins and cloned it into a GFP expression vector. Our initial assays adopted an mCherry reporter system. Briefly, we introduced stop codons (G‐to‐A mutation) in the mCherry coding sequence (affecting W63, W98, or W148), only when a fusion protein achieves an A‐to‐I conversion of the mutated position of the substrate reporter mRNA, the red fluorescence signal (RFP) will be evident (Figure 1b). We transfected human embryonic kidney (HEK) 293T cells with Cas‐ADAR^RESCUE‐S^ fusion protein, guide RNA, and reporter plasmid and then quantified levels of targeted A‐to‐I conversion 48 h later. All of the examined fusion proteins, with the exception of the SsCas6 fusion protein, restored RFP signal to some extent at the three sites, supporting successful RNA base editing (Figure 1c). Among them, PaCsf5, EcCas6e and MmCas6 proteins showed higher editing efficiency, while MmCas6 protein showed high transcriptome‐wide off‐target (Figure 1d). Taking into account the efficiency and off‐target effect, we subsequently selected EcCas6e protein, which has been applied to track RNA distribution in living cells,^[^ ^11^ ^]^ for follow‐up exploration. Altogether, these observations indicate that small proteins from Class 1 CRISPR family, besides the classical Cas13 family, can mediate RNA base editing by binding to specific RNA deaminases.

### 2.2 ceRBE, An Optimized EcCas6e^H20L^ Fusion Editor, Exhibits High Editing Efficiency and Low Off‐Target Edits in HEK293T Cells

**Figure 2:**
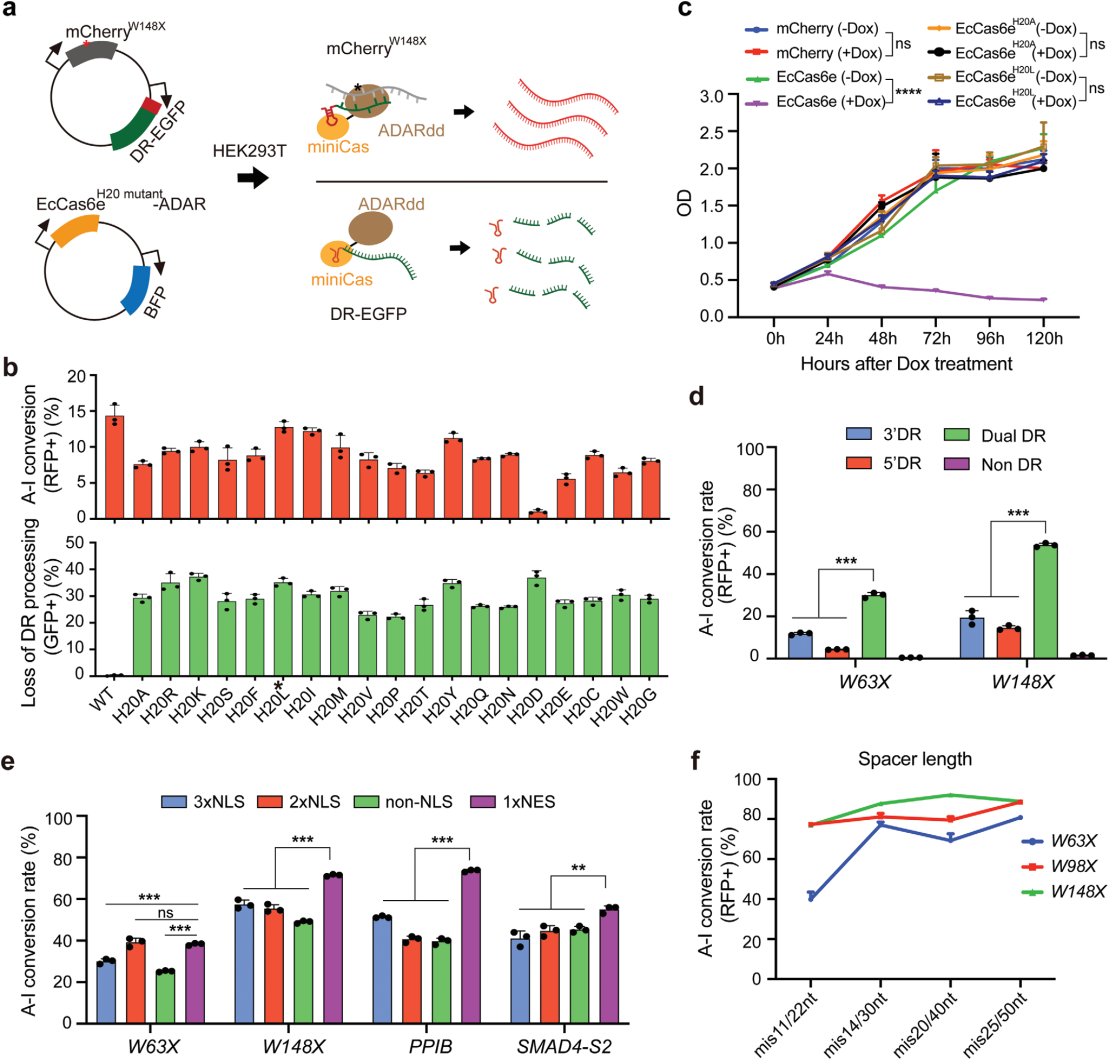
Optimization for an efficient and less off‐target base editor, ceRBE, in HEK293T. a) Schematic of screening EcCas6e H20 mutants with high efficiency and no DR processing ability. The reporter contains an mCherry CDS with a stop codon at W148 (TGG to TAG) and a EGFP transcript harboring a DR sequence between its ATG and CDS sequence initiated by the cytomegalovirus (CMV) promoter, respectively. Red fluorescence (RFP) can only be detected if the base editing is successful. Only when the protein loses DR processing function, green fluorescent protein (GFP) can be expressed. b) Flow cytometry results of EcCas6e H20 mutants. The higher the RFP ratio, the higher the efficiency of A‐to‐I conversion. The higher the ratio of GFP, the better the disruption of DR processing ability. The H20L mutant, marked with an asterisk, is chose later. c) MTT assay for the stable cell line expressing mCherry, EcCas6e, EcCas6e^H20A^and EcCas6e^H20L^ protein with and without Doxorubicin (Dox) treatment. mCherry is as a control. OD, optical density. All values are presented as means ± SD (n = 5). ^****^P < 0.0001; ns, no statistically significant. Tukey's multiple comparisons test was used after one‐way ANOVA. d–f) Base editing efficiency of EcCas6e^H20L^‐mediated editors with different guide RNA components (d), different subcellular localization sequences (e), and different spacer lengths (f). The ratio of red fluorescence or Sanger sequencing data is used to indicate editing efficiency. PPIB and SMAD4 are genes expressed in HEK293T cells. All values of d‐f are presented as means ± SD (n = 3); Statistical analysis was calculated by Student's *t*‐test; ns, no statistically significant. ^**^ *p*<0.01, ^***^ *p*<0.001. See Supporting Information Data 2 for detailed data.

Then, we optimized the EcCas6e protein for toxicity and editing efficiency. We found EcCas6e expression cell lines generated by piggyBac transposon showed greatly reduced growth rates, indicating cytotoxicity of EcCas6e (**Figure** **2**c; Figure S1, Supporting Information). Given a previous report that mutating H (His) 20 residue of EcCas6e to A (Ala) can disrupt its DR processing function,^[^ ^23^ ^]^ we examined the variant editor bearing this mutation and detected obviously reduced cytotoxicity (Figure 2c). We additionally performed saturation mutagenesis of the EcCas6e protein at H20 site and conducted assays using the mCherry reporter mentioned above and the EGFP transcript harboring a DR sequence between its ATG and CDS sequences. In these cells, EGFP fluorescence is only evident when the DR processing function of an examined EcCas6e variant is lost, and RNA base editing efficiency can still be monitored through the RFP fluorescence signal (Figure 2a). Flow cytometry showed that all mutants of the H20 site disrupted DR processing function of EcCas6e. Among them, the editors based on the H20L and H20I EcCas6e variants showed the highest editing efficiency (Figure 2b). Cell line expressing EcCas6e^H20L^ variant with severe loss of DR processing capacity also did not induce any significant cytotoxicity (Figure 2c).

Previous reports have shown that different Cas13 proteins and their mediated editors have different preferences for crRNA architecture, subcellular localization and spacer length.^[^ ^1^, ^2^, ^3^ ^]^ Therefore, in order to further improve the efficiency of base editing, we next optimized the EcCas6e^H20L^ fusion editor for these aspects. We first compared the effects of DR position at the 5ʹ end, 3ʹ end or both ends of crRNA using an mCherry fluorescent reporter. The crRNA with both 5’ and 3ʹ DR (dual DR) showed the highest red fluorescence ratio (Figure 2d). For subcellular localization, we inserted the EcCas6e^H20L^ fusion editor into a vector with three or two nuclear localization sequences (NLS) or one HIV Rev nuclear export sequence (NES) or a vector without any localization signal. We tested guide RNA targeting the mCherry reporter gene or endogenous genes (PPIB; SMAD4‐S2, SMAD4 site 2), respectively, and found that editor fused to the NES showed comparable or highest efficiencies (Figure 2e and Table S2, Supporting Information). In order to determine the optimal spacer length, we uniformly tiled guide RNAs with spacers 22, 30, 40, or 50 nucleotides (nt) long across the target adenosine. All guide lengths were functional, with 50 nt being relatively optimal (Figure 2f and Table S2, Supporting Information). Hereafter, the optimized compact and efficient RNA Base Editor mediated by EcCas6e^H20L^ protein is referred to as ceRBE.

**Figure 3:**
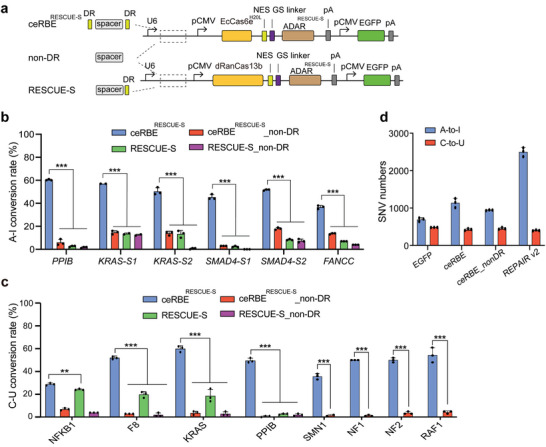
Efficient and specific base editing activity of ceRBE in HEK293T cells. a) Schematic diagram of plasmids. b,c) The A‐to‐I (b) or C‐to‐U (c) editing efficiency of the ceRBE editor, with the deaminase from the RESCUE‐S editor, and RESCUE‐S editor on endogenous transcripts in HEK293T analyzed with Sanger sequencing. In the previous article^[^ ^3^ ^]^ the RESCUE‐S editor showed no effect at SMN1, NF1, NF2 and RAF1 sites, so they were not explored here. non‐DR, guide RNA without DRs, is as a control. d) Transcriptome‐wide off‐target sites numbers for EGFP (control), ceRBE, ceRBE_nonDR and REPAIR v2 transfection experiments in HEK293T cells. Guide RNA targets SMAD4 site 2 (SMAD4‐S2). All values are presented as means ± SD (n = 3 or n = 2); Statistical analysis was calculated by Student's t‐test; ^**^ *p*<0.01, ^***^ *p*<0.001. See Supporting Information Data 3 for detailed data.

To confirm that ceRBE is broadly applicable as previously reported dCas13‐meditated editors, we designed a number of crRNAs for endogenous sites to explore. We found crRNA‐dependent A‐to‐I conversion or C‐to‐U conversion were efficiently achieved by ceRBE but not RESCUE‐S containing the same deaminase (**Figure** **3**a–c: Figures S4 and S5 and Table S3, Supporting Information), confirming the results with reporter assay. It was worth noting that ceRBE also achieved very efficient editing at the SMN1, NF1, NF2, and RAF1 sites that cannot be edited by RESCUE‐S in the previous article (Figure 3c), and was even generally better than the base editing system in the previous article.^[^ ^3^ ^]^ Moreover, ceRBE exhibited lower transcriptome‐wide off‐target effects in contrast with the REPAIR editor with the same deaminase, indicating its safety (Figure 3d; Figure S2, Supporting Information).

### 2.3 Restoration of Dystrophin Expression in the DMD^Q1392X^ Humanized Mice by AAV Delivery of ceRBE

Duchenne muscular dystrophy (DMD) is the second most common hereditary muscular disease, because of the deficiency of dystrophin (DMD), affecting an estimated 1 in 3500–5000 male newborns.^[^ ^24^ ^]^ The Q1392X (c.4174C>T) mutation, which we identified in the DMD gene on chromosome X of a patient with progressive myasthenia, results in the loss of dystrophin in multiple muscle tissues. To explore the therapeutic effect of ceRBE at this disease site, we fused the human exon 30 coding sequence (CDS) containing the Q1392X mutation with mCherry via T2A (self‐cleaving peptide) sequence to overexpress DMD^Q1392X^ RNA in HEK293T cells (Figure S3a, Supporting Information). After co‐transfecting with gRNAs and the ceRBE with hADAR2dd^E488Q^ protein, which shows highest efficiency among adenine deaminases, we found that gRNAs carrying mismatch nucleotide against Q1392X mutation on different positions achieved editing efficiencies of 72.5% to 85.7% (Figure S3b and Table S4, Supporting Information). Taking into account the efficiencies of on‐target and bystander off‐targets, we subsequently selected the gRNA with the tenth mismatch nucleotide.

**Figure 4:**
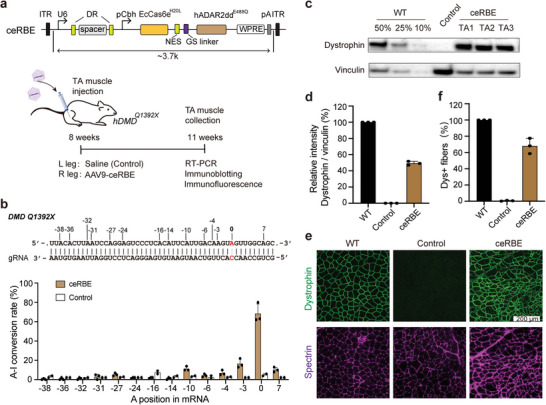
ceRBE successfully rescues dystrophin expression in humanized DMD^Q1392X^ mice after AAV delivery. a) Schematic diagram of the in vivo intramuscular (IM) injection of the AAV9‐ceRBE in the TA muscle of the right leg of 8‐week‐old humanized DMD^Q1392X^ mice. Left leg was injected with saline as a control. TA muscle was harvested 3 weeks after intramuscular (IM) injection. b) The A to I conversion rate of AAV9‐ceRBE vector. A cDNA amplicon spanning exon 30 was generated from the TA muscle and analyzed by Sanger sequencing. Targeting site number is 0. Others are sites with bystander editing, with “‐’on the left and “+” on the right which was omitted. Numbers represent the distance from the targeting site. n = 3 (ceRBE) or 2 (Control). c) Immunoblotting analysis of dystrophin (Sigma, D8168) and vinculin (CST, 13901S) expression in TA muscles 3 weeks after injection with AAV9‐ceRBE. WT, normal C57BL/6J mouse. d) Quantification of dystrophin expression from immunoblotting after normalization to vinculin expression. WT, normal C57BL/6J mouse. Saline‐treated legs were as controls. (e,f) Immunofluorescence analysis. No dystrophin signal was detected in saline‐treated control. Dystrophin (Abcam, ab15277) is shown in green. Spectrin (Millipore, MAB1622) is shown in magenta. Scale bars, 200 µm (e). Quantification of positive dystrophin (Dys+) fibers in total spectrin‐positive myofibers in cross sections of TA muscles (f). The data are presented as the means values ± SD (n = 3). See , Supporting Information Data 4 for detailed data.

For in vivo treatment, we constructed a humanized mouse model of DMD, in which the human DMD exon30 carrying the Q1392X mutation replaces the corresponding mouse Dmd exon 30 (Figure S3c and Tables S4 and S5, Supporting Information). ceRBE with hADAR2dd^E488Q^ protein and gRNA with the tenth mismatch nucleotide were packaged into single AAV9 particle. The right tibialis anterior (TA) of 8‐week‐old male DMD^Q1392X^ mice was injected with 5 × 10^11^ vg of AAV9 viral vehicles, while the left TA of the same mice were injected with saline as a control (**Figure** **4**a). At 3 weeks post injection, we harvested TA tissue from the injected DMD^Q1392X^ mice for analysis of base editing efficiency, immunoblotting, and immunofluorescence for dystrophin expression (Figure 4a). Sequencing data revealed that the editing efficiency of A‐to‐I reached 68.3±10.1% in TA tissue treated with AAV9 (Figure 4b). Immunoblotting indicated that the expression of dystrophin protein was also largely restored in AAV9‐treated DMD mice (49.3 ± 2.4%) (Figure 4c,d). To quantify the efficiency of dystrophin rescue in TA tissue, we performed histological staining and examined dystrophin and spectrin (internal reference) expression. The results showed that dystrophin was rescued to a high level (68.1 ± 9.4%) in TA tissues after virus delivery (Figure 4e,f), which was consistent with the immunoblotting results of tissues.

We further explored the off‐target effects of ceRBE in vivo. We collected RNA from saline‐injected and ceRBE‐injected TA tissues for RNA sequencing and SNV number analysis. Compared to saline control, the number of SNVs was not significantly increased in the ceRBE‐treated group (Figures S6 and S7, Supporting Information), suggesting that ceRBE is specific in vivo. Overall, these results demonstrate the potential of ceRBE for efficient RNA editing in vivo, especially for certain genetic diseases.

## 3 Discussion

In sum, we propose a novel RNA base editor–ceRBE with a small size and high in vivo editing efficiency. The ceRBE editor achieved A‐to‐I base editing efficiencies of 68.3±10.1% in DMD^Q1392X^ mice using AAV‐mediated delivery, and successfully restored the expression of dystrophin protein (Figure 4).

It has to be noted that that our study found that, except for the EcCas6e protein, several other small proteins also achieved base editing to varying degrees (Figure 1c). It is inferred that other small pre‐crRNA processing proteins also have the potential to replace dCas13 proteins to achieve base editing, greatly expanding the selection range of Cas proteins in RNA base editors. In addition, EcCas6e^H20L^ protein can also be applied to technologies such as RNA degradation and live RNA imaging, which can be mediated by the Cas13 protein.^[^ ^25^ ^]^

Compared with the systems based on Cas13/crRNA complex targeting RNA, the Cas6‐derived RNA editors only use spacer instead of Cas6 to target RNA, and the spacer content may inhibit the stability of DR hairpin motif. Therefore, it may be beneficial to consider the secondary structure of DR‐spacer‐DR in the gRNA design process to improve the efficiency of RNA editing.

In the exploration, we found that the double DR strategy outperformed the single DR strategy for the EcCas6e^H20L^ editor that lost the DR processing ability (Figure 2d). We speculate that the double DR structure may make the crRNA more stable and can also recruit more EcCas6e^H20L^ editors than single DR structure, thereby improving the editing efficiency. Notably, the double DR strategy may also be applicable to other RNA editing tools that lack DR processing capabilities.

Beyond Duchenne muscular dystrophy, it should be straightforward to apply ceRBE editor to treat other diseases caused by G‐to‐A mutation using AAV‐mediated delivery. However, it bears mention that the ceRBE editor does elicit slightly more bystander off‐target editing compared with the control (Figure 4b; Figures S3b, S4 and S5, Supporting Information). Thus, to further support the clinical application prospects of this editor, additional optimization, such as the introduction of bulges at all bystander‐edited adenosines,^[^ ^26^ ^]^ is warranted.

## 4 Experimental Section

### Plasmid Constructions

Coding sequence of different small Cas proteins or EcCas6e H20 mutants were human‐codon‐optimized and flanked with SV40 NLS (CCTAAGAAGAAGCGGAAGGTG) or HIV NES (CTGCCTCCACTTGAAAGAC TGACACTG). And then it was fused to the ADAR protein, the deaminase in the RESCUE‐S editor, or hADAR2dd^E488Q^ via a GS linker to constitute the different editing systems. They were all expressed by broad‐spectrum promoters (CMV promoter was used in HEK293T cells in vitro, and Cbh promoter was used in viruses in vivo). In vitro, EGFP or BFP fluorescence was also co‐expressed with the editing system to indicate successful transfection and expression of the vector in host cells. We synthesized different spacers as oligos and cloned them downstream of the U6 promoter to express different gRNA configurations in cells.

For fluorescent reporter system, in addition to plasmid of the base editing system mentioned above, an mCherry (red fluorescent protein, RFP) reporter system containing an early stop codon TAG mutated from TGG codon (W) at W63 or W98 or W148 in its mCherry coding sequence also need to be co‐transformed.

When DMD^Q1392X^ were explored in vitro, reporter vectors were constructed using mCherry and ATG‐deleted human exon 30 sequences carrying the Q1392X (c.4174C>T) mutation. mCherry fluorescence indicated successful transfection and expression of the reporter vector in host cells.

All primers for PCR, sequence of proteins and gRNAs used in this study are provided in Tables S1–S5 (Supporting Information).

### Cell Culture, Transfection, and Flow Cytometry Analysis

HEK293T, a mammalian cell line, was used in the study. It was incubated at 37°C under 5% CO2 in a humidified incubator. Medium consisted of DMEM containing 1% penicillin/streptomycin, 10% fetal bovine serum, and 1% non‐essential amino acids. For fluorescence detection, HEK293T was passaged at a ratio of 1:3 to 12‐well plates. After 16 h, 1 µg plasmid expressing base editor and 1 µg fluorescent reporter plasmid were co‐transfected with polyethylenimine (PEI) using the standard protocol. 48 h after transfection, cells were analyzed by Beckman cytoFLEX. FlowJo X (v.10.0.7) were used for analyze Flow cytometry results.

For endogenous sites, HEK293T was passaged at a ratio of 1:3 to 6‐well plates. After 16 h, 3 µg plasmid expressing base editor was transfected into cells with polyethylenimine (PEI) using the standard protocol. Cells were sorted by BD FACSAria III at 48 h after transfection.

For gRNA screening of DMD^Q1392X^, HEK293T was passaged at a ratio of 1:3 to 6‐well plates. After 16 h, 1.5 µg CRISPR targeting plasmids and 1.5 µg reporter were co‐transfected with polyethylenimine (PEI) using the standard protocol. Cells were sorted by BD FACSAria III at 48 h after transfection.

### MTT Assay

Briefly, single‐cell clones with mCherry, EcCas6e, EcCas6e^H20A^ and EcCas6e^H20L^ were plated on a 96‐well plate at 5000 cells per well and were treated with or without Doxorubicin (Dox) (1 µg ml^−1^) for 0, 24, 48, 72, 96, or 120 h. Then, 10 µL of freshly prepared MTT (5 mg mL^−1^, Beyotime; Catalog # C0009M) was added to each well. After 3 h incubated with MTT, 100 µL of dissolved formazan liquid (Beyotime; Catalog # C0009M) was added into 96‐well plate for 4 h at 37°C. Absorbance was measured at 570 nm by a microplate reader (Bio TeK cytation).

### RT‐PCR and Sequencing Analysis

To analyze base conversion rate of different base editors, transfected positive cells were sorted 48 h after transfection for RNA extraction at. According to the manufacturer protocol, total RNA of sorted cells or isolated tissues were isolated and converted to cDNA using the reverse transcription kit (HiScript Q RT SuperMix for qPCR, Vazyme, Biotech, P611‐01). Targeted editing sites were amplified using Phanta Max Super‐Fidelity DNA Polymerase (Vazyme, P505‐d1) for Sanger sequencing or deep sequencing. Sanger sequencing results were analyzed using EditR 1.0.10^[^ ^27^ ^]^ to quantify base conversion rate. Deep sequencing libraries were sequenced with 150 paired‐end reads on an Illumina Hiseq instrument. FASTQ format data were analyzed using the Cutadapt (v.2.8) according to assigned barcode sequences.

All primers for PCR used in this study are provided in Table S5 (Supporting Information).

### RNA Sequencing Analysis

RNA‐seq reads were trimmed and edit sites were identified as described previously^[^ ^3^, ^28^ ^]^ For HEK293T, SNPs filtering was done using the database downloaded from NCBI, 1000 Genomes Project (https://www.internationalgenome.org/) and the University of Washington Exome Sequencing Project (https://evs.gs.washington.edu/EVS/). For in vivo, the mouse mm10 genome and dbSNP of ensemble version 104 (https://www.ensembl.org/) were used to calculate and filter SNVs respectively.

### Generation of DMD^Q1392X^ Mouse Model

DMD^Q1392X^ mice were generated using the CRISPR‐Cas9 system as in reference.^[^ ^29^ ^]^ Mouse Dmd exon 30 was deleted by CRISPR‐Cas9 guided by two sgRNAs flanking exon 30 (Table S4, Supporting Information), and replaced with human exon 30 carried nonsense mutation (Figure S3c, Supporting Information). All experimental protocols were approved by China and HuidaGene Therapeutics Co., Ltd.

### AAV9 Production and Delivery to DMD^Q1392X^ Mice

AAVs were produced by PackGene Biotech (Guangzhou, China), and applied iodixanol density gradient centrifugation for purification. For intramuscular injection, 8‐week‐old male DMD^Q1392X^ mice were anesthetized, and the right TA (tibialis anterior) muscle was injected with 50 µL of AAV9 (5 × 10^11^ vg) viral vehicles, while the left TA of the same mice were injected with same volume saline solution as a control (Figure 4a).

### Immunoblotting

Isolated tissues were homogenized with RIPA buffer supplemented with protease inhibitor cocktail. Adjust lysate supernatants to an identical concentration using H2O, after quantifying it with Pierce BCA protein assay kit (Thermo Fisher Scientific, 23 225).

For DMD^Q1392X^ mice, equal amounts of samples were mixed with in NuPAGE LDS sample buffer (Invitrogen, NP0007) and 10% *β*‐mercaptoethanol and then were boiled at 70°C for 10 min. 10 µg total protein per lane was loaded into 3 to 8% tris‐acetate gel (Invitrogen, EA03752BOX) and electrophoresed for 1 h at 200 V. Protein was transferred to a PVDF membrane under wet condition at 350 mA for 3.5 h. The PVDF membrane was incubated with primary antibody labeling specific protein after blocking in 5% non‐fat milk in TBST buffer. The membrane was further incubated with HRP conjugated secondary antibody specific to the IgG of the species of primary antibody against dystrophin (Sigma, D8168) and vinculin (CST, 13901S), after washing three times with TBST. The target proteins were visualized with Chemiluminescent substrates (Invitrogen, WP20005). The gray values were quantified by using ImageJ.

### Immunofluorescence (IF)

The mice were anesthetized and perfused with PBS solution and subsequent 4% paraformaldehyde (PFA) solution. The TA muscles of the mice were harvested and fixed in 4% PFA. After 8 h, the tissues were washed and dehydrated with PBS and 30% sucrose, respectively. Subsequent cryosection and staining of TA muscle tissue were performed as in reference.^[^ ^29^ ^]^ All images were visualized under Nikon C2. The amount of dystrophin positive muscle fibers is represented as a percentage of total spectrin positive muscle fibers.

### Statistical Analysis

All data were shown as means ± standard deviation (SD). Unless otherwise mentioned, n = 3 independent experiments for in vitro and in vivo experiments. Tukey's multiple comparisons test and two‐tailed paired Student's t‐test were used to calculate significance levels. The significance levels are ns (*p* > 0.05), ^*^ (*p* < 0.05), ^**^ (*p*<0.01), ^***^ (*p*<0.001) and ^****^ (*p*<0.0001), compared with control. GraphPad Prism v8.0 software was used for statistical analysis.

## Conflict of Interest

Y.Z., H.Y., X.W., and R.Z. disclosed a patent applications related to this work. H.Y. is the founder of HuidaGene Therapeutics Co., Ltd. The remaining authors declare no competing interests.

## Author Contributions

X.W., R.Z., H.Y., and Y.Z. jointly conceived the project. H.Y. and Y.Z. supervised the whole project. X.W., R.Z., D.Y., and G.L. designed and conducted experiments. X.W. and R.Z. designed vectors, performing in vitro experiments. G.L. designed and completed DMD^Q1392X^ exploration. Z.F. and H.D. assisted with plasmids construction and cell experiments. J.L. and XQ.W. assisted with in vivo exploration. Y.Z. and Y.L. conducted bioinformatics analysis. Z.W. and L.S. assisted in experimental discussion. X.W. and R.Z. analyzed the data and organized figures. X.W., D.Y., R.Z., and Y.Z. wrote the manuscript with help from by all authors.

## Supporting information

Supporting Information

Supporting Information

Supporting Information

## Data Availability

The underlying data for this article are provided in this paper and its online , Supporting Information Data. Raw RNA‐seq data have been deposited in NCBI SRA (accession number: PRJNA898585 and PRJNA935253).
