# Supplementary material for: Develop a Compact RNA Base Editor by Fusing ADAR with Engineered EcCas6e
Source: Adv Sci (Weinh). 2023 Apr 25;10(17):2206813. doi: 10.1002/advs.202206813 (PMC10265090; doi:10.1002/advs.202206813)
Supplement: Supplementary file 1 — Supporting Information [file ADVS-10-2206813-s002.pdf]

ADVANCED SCIENCE
Open Access

Supporting Information

for *Adv. Sci.*, DOI 10.1002/advs.202206813

Develop a Compact RNA Base Editor by Fusing ADAR with Engineered EcCas6e

Xing Wang, Renxia Zhang, Dong Yang, Guoling Li, Zhanqing Fan, Hongting Du, Zikang Wang, Yuanhua Liu, Jiajia Lin, Xiaoqing Wu, Linyu Shi, Hui Yang and Yingsi Zhou**

Supplementary Figures

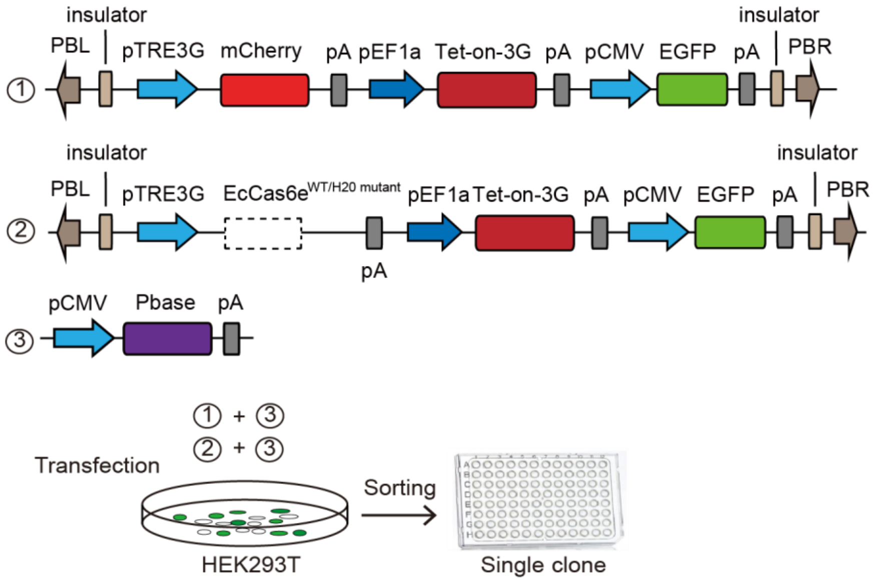

Supplementary Figure 1.

Cell lines generated using *piggyBac* transposon.

Schematic diagram of *piggyBac* transposon generation of Dox-inducible cell lines stably expressing EcCas6e, EcCas6e^{H20A}, EcCas6e^{H20L} or mCherry in HEK293T cells.

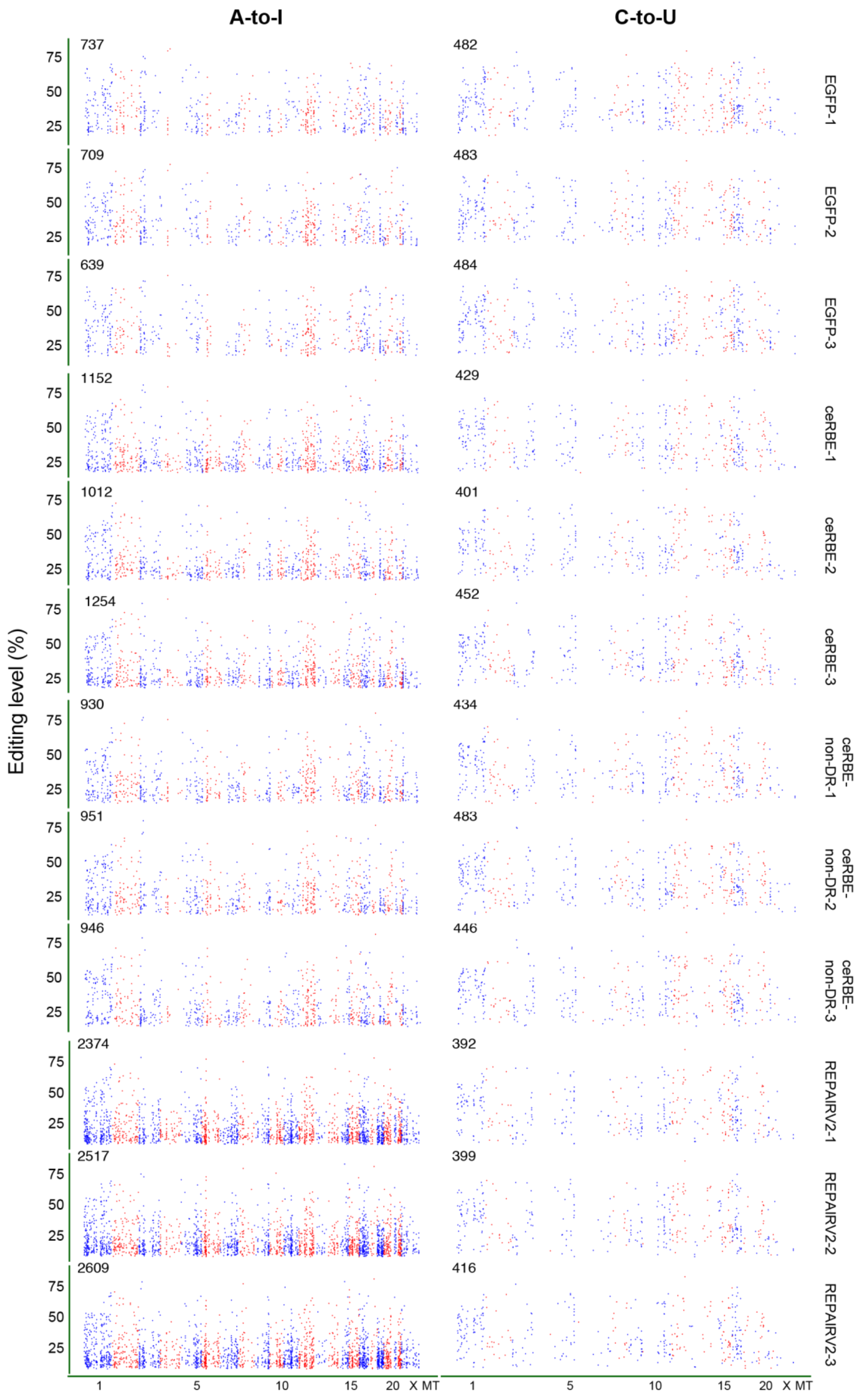

Supplementary Figure 2.

Manhattan plots of transcriptome-wide off-target RNA editing.

Analysis for EGFP (control), ceRBE, ceRBE_nonDR and REPAIR v2 transfection experiments in HEK293T cells. A-to-I editor targeting endogenous *SMAD4* RNA; The x and y axes are proportionally enlarged with each Manhattan plot to make the axis legend clear. nonDR, guide RNA without DRs. The data are presented by the values of each biological repeat experiment (n = 3).

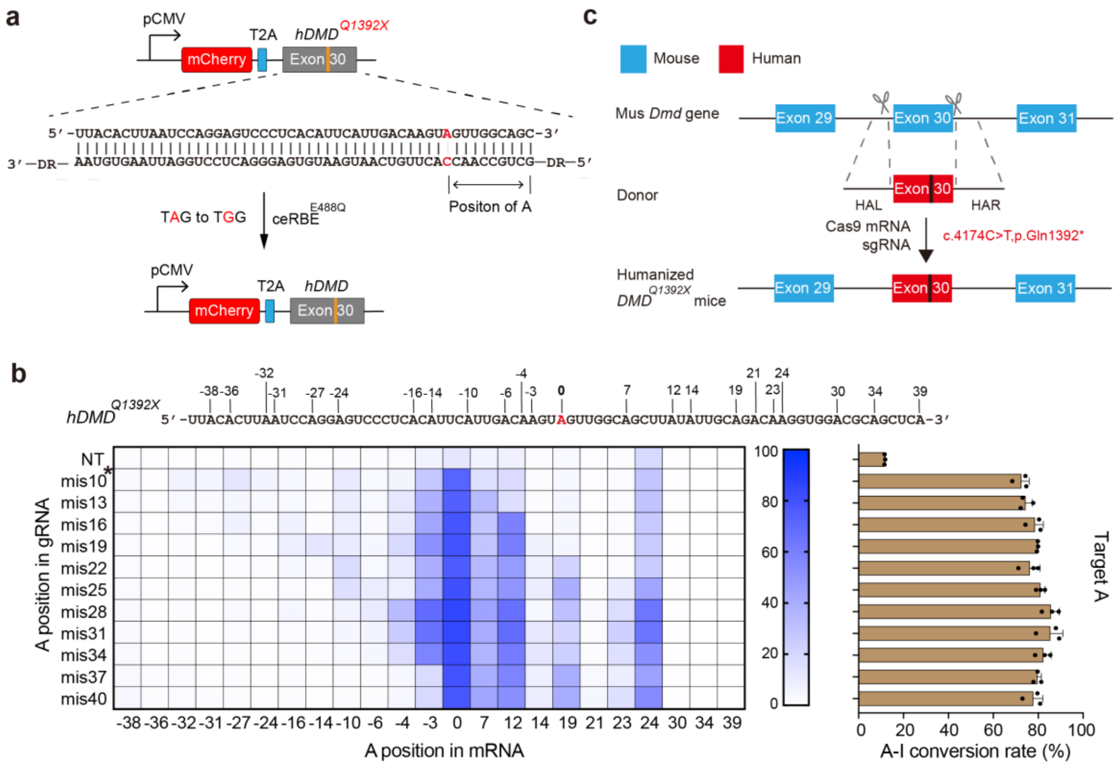

Supplementary Figure 3.

Editing efficiency of the Q1392X site on *DMD* gene in HEK293T.

(a) Schematic diagram of the reporting system for exploring editing efficiency of *DMD*^{Q1392X} in HEK293T. The reporter construct contains the mCherry cassette fused with 2A peptide, and mutant human exon 30 (p.Q1392X). ‘A’ marked in red, targeting site. (b) Measurement of bystander A to I editing rate for multiple adenosines within targeting sequence of all gRNA from deep-sequencing. ‘A’ marked in red, targeting site. Targeting site number is 0. Others are sites with bystander editing, with ‘-’on the left and ‘+’ on the right which was omitted. Numbers represent the distance from the targeting site. The A-to-I conversion rates of the targeting site are also listed separately on the right. Mis, Mismatch. NT, non-targeting crRNA. Asterisks, representing subsequent selected group. All values are presented as means ± SD (n=3). (c) Strategy for generation humanized *DMD*^{Q1392X} mice. Mouse DMD exon 30 was deleted by CRISPR-Cas9 guided by two gRNAs flanking exon 30, and replaced with human exon 30 carried nonsense mutation. All values are presented as means (n=3). See Supplementary Data 5 for detailed data.

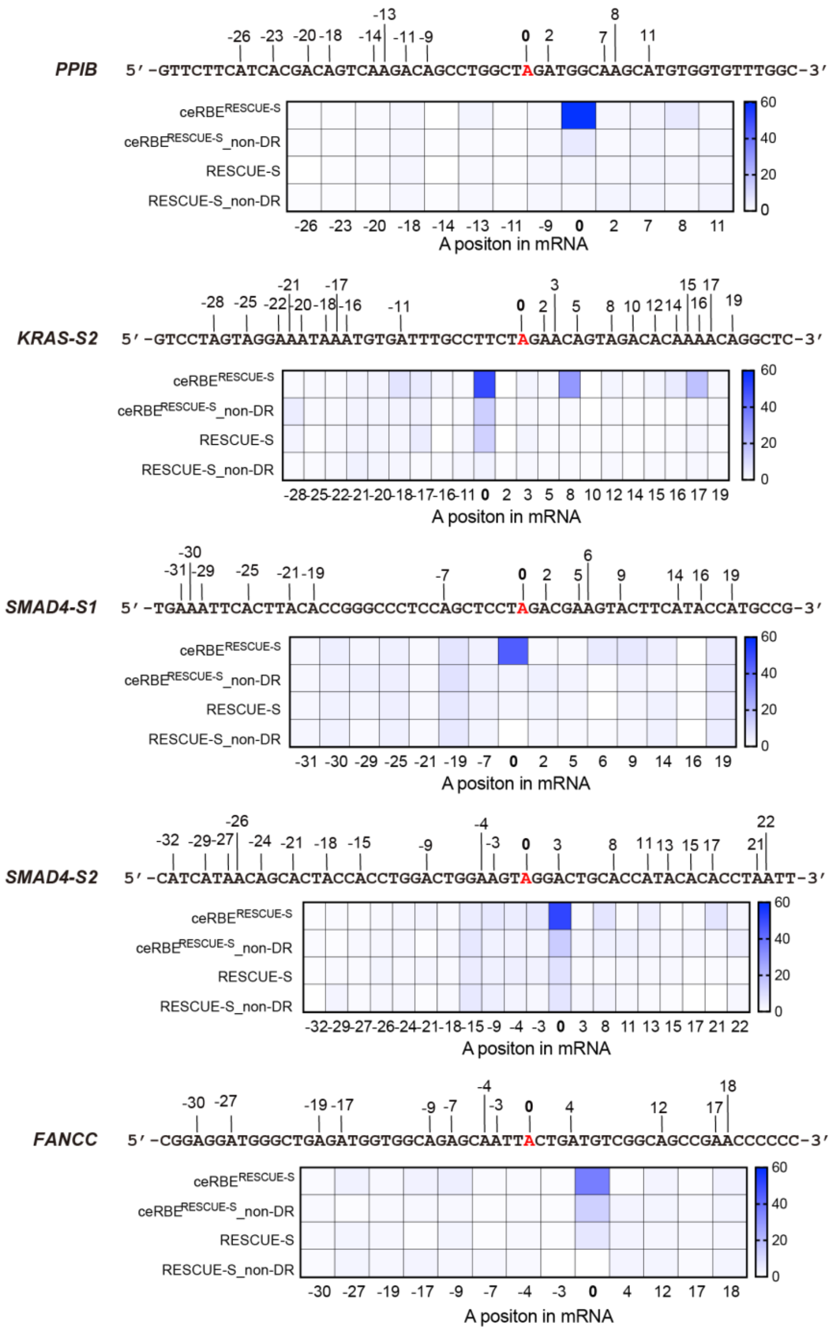

Supplementary Figure 4.

Bystander editing rates of multiple adenosines in endogenous targeting sequences for A-to-I conversion in HEK293T.

Corresponding to Figure 3b. ‘A’ marked in red, targeting site. Targeting site number is 0. Others are sites with bystander editing, with ‘-’on the left and ‘+’ on the right which was omitted. Numbers represent the distance from the targeting site. All values are presented as means (n=3). See Supplementary Data 6 for detailed data.

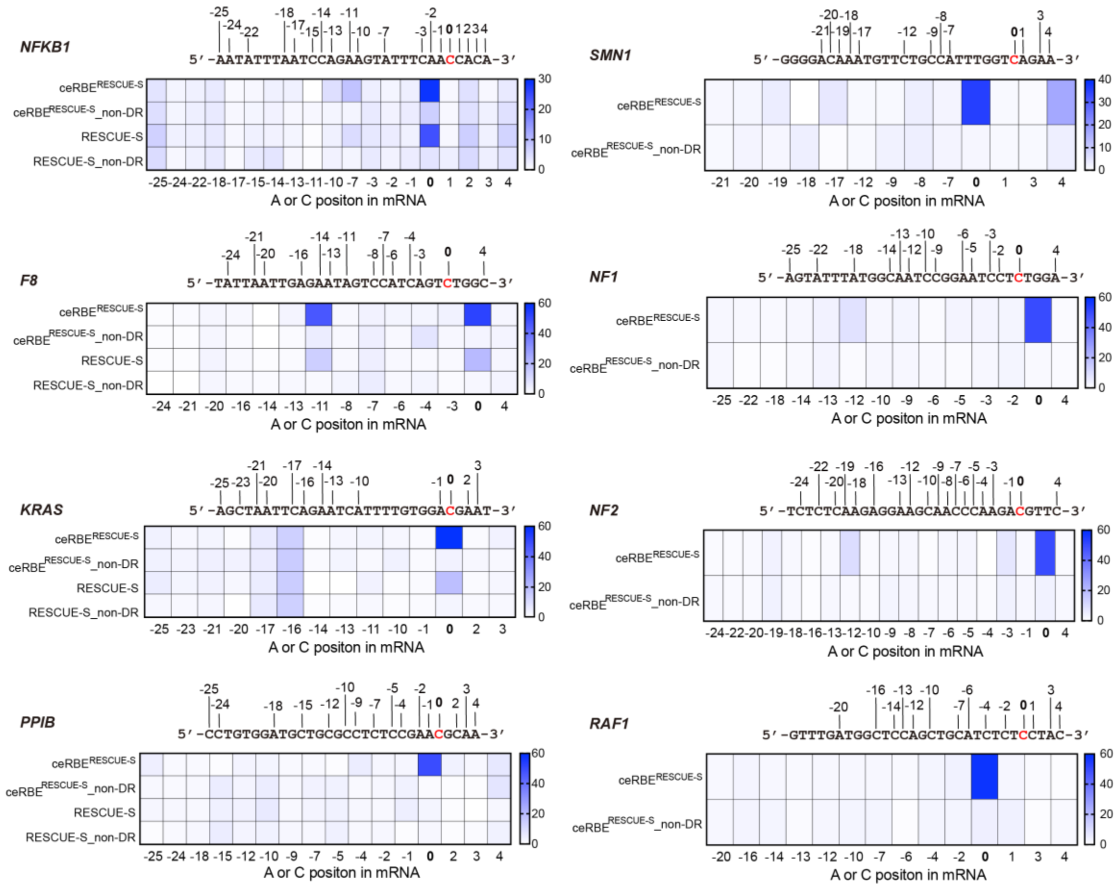

Supplementary Figure 5.

Bystander editing rates of multiple adenosines and cytosines in endogenous targeting sequences for C-to-U conversion in HEK293T.

Corresponding to Figure 3c. ‘C’ marked in red, targeting site. Targeting site number is 0. Others are sites with bystander editing, with ‘-’on the left and ‘+’ on the right which was omitted. Numbers represent the distance from the targeting site. All values are presented as means (n=3 or 2). See Supplementary Data 7 for detailed data.

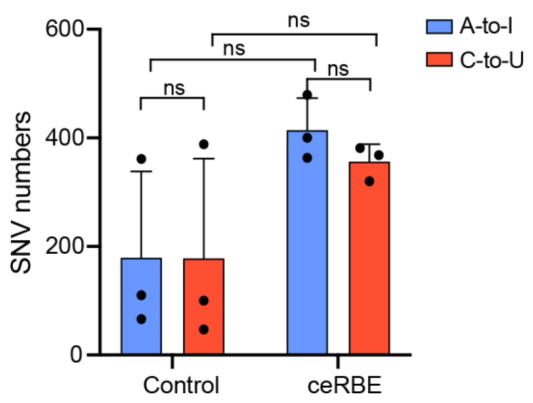

Supplementary Figure 6.

Calculation of SNV numbers from transcriptome-wide off-target analysis *in vivo*.

The transcriptome-wide off-target effects of ceRBE after AAV injection were analyzed. Control, only saline was injected. Data are shown as means values ± SD (n=3), and are compared using unpaired Student's t-test (two-tailed). ns, no statistically significant. See Supplementary Data 8 for detailed data.

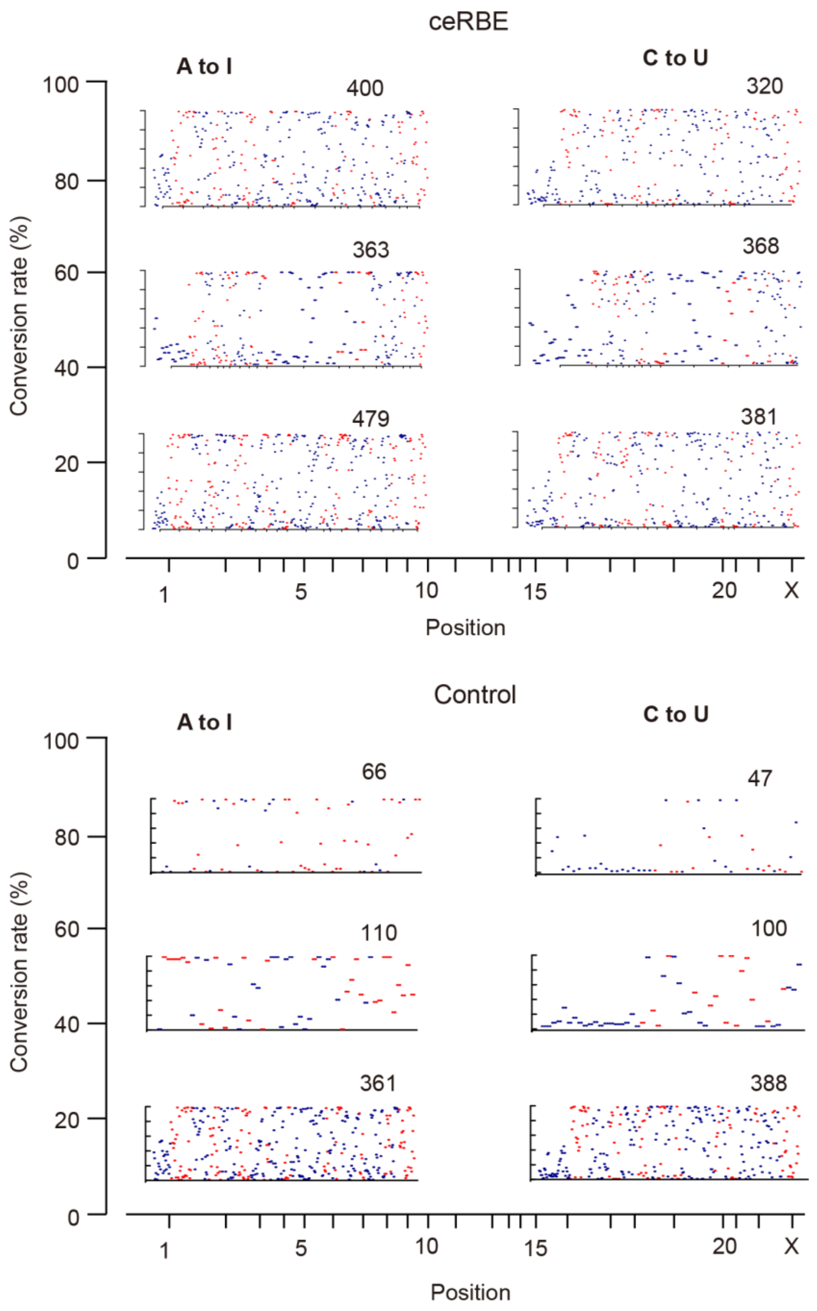

Supplementary Figure 7.

Manhattan plots of transcriptome-wide off-target RNA editing *in vivo*.

Corresponding to Supplementary Figure 6. The x and y axes are proportionally enlarged with each Manhattan plot to make the axis legend clear. The data are presented by the values of each biological repeat experiment (n = 3).
