# Supplementary material for: Develop a Compact RNA Base Editor by Fusing ADAR with Engineered EcCas6e
Source: Adv Sci (Weinh). 2023 Apr 25;10(17):2206813. doi: 10.1002/advs.202206813 (PMC10265090; doi:10.1002/advs.202206813)
Supplement: Supplementary file 2 — Supporting Information [file ADVS-10-2206813-s003.pdf]

ADVANCED SCIENCE
Open Access

Supporting Information

for *Adv. Sci.*, DOI 10.1002/advs.202206813

Develop a Compact RNA Base Editor by Fusing ADAR with Engineered EcCas6e

Xing Wang, Renxia Zhang, Dong Yang, Guoling Li, Zhanqing Fan, Hongting Du, Zikang Wang, Yuanhua Liu, Jiajia Lin, Xiaoqing Wu, Linyu Shi, Hui Yang and Yingsi Zhou**

Supplementary Tables

Supplementary Table 1.

Ten representative Cas proteins from Class 1 CRISPR proteins.

**Subtype**	**Protein**	**Species**	**DR sequence**	**Size**	**Amino acid sequence**
I-A	SsoCas6	Sulfolobus solfataricus	GATAATCTCTTATAGAATTGAAAG	289aa	MPLIFKIGYNVIPLQDVILPTPSSKVLKYLIQSGKLIPSLKDLITSRDKYKPIFISHLGFNQRRIFQTNGNLKTITKGSRLSSIIAFSTQANVLSEVADEGIFETVYGKFHIMIESIEIVEVEKLKEEVEKHMNDNIRVRFVSPTLLSSKVLLPPSLSERYKKIHAGYSTLPSVGLIVAYAYNVYCNLIGKKEVEVRAFKFGILSNALSRIIGYDLHPVTVAIGEDSKGNLRKARGVMGWIEFDIPDERLKRRALNYLLTSSYLGIGRSRGIGFGEIRLEFRKIEEKEG
I-B	MmCas6	Methanococcus maripaludis	CTAAAAGAATAACTTGCAAAATAACAAGCATTGAAAC	218aa	MDLEYMHISYPNILLNMRDGSKLRGYFAKKYIDEEIVHNHRDNAFVYKYPQIQFKIIDRSPLIIGIGSLGINFLESKRIFFEKELIISNDTNDITEVNVHKDMDHFGTTDKILKYQFKTPWMALNAKNSEIYKNSDEIDREEFLKRVLIGNILSMSKSLGYTIEEKLKVKINLKEVPVKFKNQNMVGFRGEFYINFDIPQYLGIGRNVSRGFGTVVKV
I-C	BhCas5d	Bacillus halodurans	GTCGCACTCTTCATGGGTGCGTGGATTGAAAT	236aa	MRNEVQFELFGDYALFTDPLTKIGGEKLSYSVPTYQALKGIAESIYWKPTIVFVIDELRVMKPIQMESKGVRPIEYGGGNTLAHYTYLKDVHYQVKAHFEFNLHRPDLAFDRNEGKHYSILQRSLKAGGRRDIFLGARECQGYVAPCEFGSGDGFYDGQGKYHLGTMVHGFNYPDETGQHQLDVRLWSAVMENG

					YIQFPRPEDCPIVRPVKEMEPKIFNPDNVQSAEQLLHDLGGE
I-C	SpCas5d	Streptococcus pyogenes	GTCTCACCCTTCATGGGTGAGTGGATTGAAAT	242aa	MYRSRDFYVRVSGQRALFTNPATKGGSERSSYSVPTRQALNGIVDAIYYKPTFTNIVTEVKVINQIQTELQGVRALLHDYSADLSYVSYLSDVVYLIKFHFVWNEDRKDLNSDRLPAKHEAIMERSIRKGGRRDVFLGTRECLGLLDDISQEEYETTVSYYNGVNIDLGIMFHSFAYPKDKKTPLKSYFTKTVMKNGVITFKAQSECDIVNTLSSYAFKAPEEIKSVNDECMEYDAMEKGEN
I-D	SsCas6	Synechococcus sp	GTTTCAGTCCCGTAGTCGGGATTTAGTGGTTGGAAAG	260aa	MPNDPYSLYSIVIELGAAEKGFPTGILGRSLHSQVLQWFKQDNPFLATELHQSQISPFSISPLMGKRHAKLTKAGDRLFFRICLLRGDLLQPLLNGIEQTVNQSVCLDKFRFRLCQTHILPGSHPLAGASHYSLISQTPVSSKITLDFKSSTSFKVDRKIIQVFPLGEHVFNSLLRRWNNFAPEDLHFSQVDWSIPIAAFDVKTIPIHLKKVEIGAQGWVTYIFPNTEQAKIASVLSEFAFFSGVGRKTTMGMGQVQVRS
I-E	EcCas6e	Enterobacteriaceae E. coli K12	GAGTTCCCCGCGCCAGCGGGGATAAACCG	199aa	MYLSKVIIARAWSRDLYQLHQGLWHLFPNRPDAARDFLFHVEKRNTPEGCHVLLQSAQMPVSTAVATVIKTKQVEFQLQVGVPLYFRLRANPIKTILDNQKRLDSKGNIKRCRVPLIKEAEQIAWLQRKLGNAARVEDVHPISERPQYFSGDGKSGKIQTVCFEGVLTINDAPALIDLVQQGIGPAKSMGCGLLSLAPL
I-F	PaCas6f	Pseudomonas aeruginosa	GTTCACTGCCGTATAGGC	187aa	MDHYLDIRLRPDPEFPPAQLMSVLFGKLHQALVAQGGDRIGVSFPDLDESRSRLGERLRIHASADD

			AGCTAAGAAA		LRALLARPWLEGLRDHLQFGEPAVVPHPTPYRQVSRVQAKSNPERLRRRLMRRHDLSEEEARKRIPDTVARALDLPFVTLRSQSTGQHFRLFIRHGPLQVTAEEGGFTCYGLSKGGFVPWF
III-A	MtCas6	Mycobacterium tuberculosis H37Rv	GTCGTCAGACCCAAAACCCCGAGAGGGGACGGAAAC	314aa	MAARRGGIRRTDLLRRSGQPRGRHRASAAESGLTWISPTLILVGFSHRGDRRMTEHLSRLTLTLEVDAPLERARVATLGPHLHGVLMESIPADYVQTLHTVPVNPYSQYALARSTTSLEWKISTLTNEARQQIVGPINDAAFAGFRLRASGIATQVTSRSLEQNPLSQFARIFYARPETRKFRVEFLTPTAFKQSGEYVFWPDPRLVFQSLAQKYGAIVDGEEPDPGLIAEFGQSVRLSAFRVASAPFAVGAARVPGFTGSATFTVRGVDTFASYIAALLWFGEFSGCGIKASMGMGAIRVQPLAPREKCVPKP
III-B	PfCas6	Pyrococcus furiosus	GTTACAATAAGACTAAAATAGAATTGAAAG	264aa	MRFLIRLVPEDKDRAFKVPYNHQYYLQGLIYNAIKSSNPKLATYLHEVKGPKLFTYSLFMAEKREHPKGLPYFLGYKKGFFYFSTCVPEIAEALVNGLLMNPEVRLWDERFYLHEIKVLREPKKFNGSTFVTLSPIAVTVVRKGKSYDVPPMEKEFYSIIKDDLQDKYVMAYGDKPPSEFEMEVLIAKPKRFRIKPGIYQTAWHLVFRAYGNDDLLKVGYEVGFGEKNSLGFGMVKVEGNKTTKEAEEQEKITFNSREELKTGV
IV	PaCsf5	Pseudomonas aeruginosa	GTATTTCCCGCGTGCGCGGGGGTGAGCGG	236aa	MFVTQVIFNIGERTYPDRARAMVAELMDGVQPGLVATLMNYIPGTSTSRTEFPTVQFGGASDGFCLLGFGDGGGAIVRDAVPLIHAALARRMPDRIIQVEHKEHSLSAEARPYVLSYTVPRMVVQKKQRHAE

					RLLHEAEGKAHLEGLFLRSLQR QAAAVGLPLPENLEVEFKGAVG DFAAKHNPNSKVAYRGLRGAVF DVNARLGGIWTAGFMLSKGYG QFNATHQLSGAVNALSE

Supplementary Table 2.

Spacer sequences used in the Figure 1 and Figure 2.

Site	Spacer	Spacer sequence	Figure
W63X	mis5/30nt	GTCCCAGGCGAAGGGCAGGGGGCCACCCTT	1c
W98X	mis5/30nt	CTCCCACTTGAAGCCCTCGGGGAAGGACAG	1c
W148X	mis5/30nt	CTCCCAGCCCATGGTTTTCTTCTGCATTAC	1c
W63X	mis11/22nt	CAGGATGTCCCAGGCGAAGGGC	2f
W63X	mis14/30nt	GGACAGGATGTCCCAGGCGAAGGGCAGGGG	2d, 2e, 2f
W63X	mis20/40nt	CTGAGGGGACAGGATGTCCCAGGCGAAGGGCAGGGGGCCA	2f
W63X	mis25/50nt	ATGAACTGAGGGGACAGGATGTCCCAGGCGAAGGGCAGGGGGCCACCCTT	2f
W98X	mis11/22nt	CACGCGCTCCCACTTGAAGCCC	2f
W98X	mis14/30nt	CATCACGCGCTCCCACTTGAAGCCCTCGGG	2f
W98X	mis20/40nt	GAAGTTCATCACGCGCTCCCACTTGAAGCCCTCGGGGAAG	2f
W98X	mis25/50nt	TCCTCGAAGTTCATCACGCGCTCCCACTTGAAGCCCTCGGGGAAGGACAG	2f
W148X	mis11/22nt	GGAGGCCTCCCAGCCCATGGTT	2f
W148X	mis14/30nt	GGAGGAGGCCTCCCAGCCCATGGTTTTCTT	2b, 2d, 2e, 2f
W148X	mis20/40nt	CCGCTCGGAGGAGGCCTCCCAGCCCATGGTTTTCTTCTGC	2f
W148X	mis25/50nt	TACATCCGCTCGGAGGAGGCCTCCCAGCCCATGGTTTTCTTCTGCATTAC	2f
PPIB	mis14/30nt	CATGCTTGCCATCCAGCCAGGCTGTCTTGA	2e
SMAD4-S2	mis14/30nt	TATGGTGCAGTCCCACTTCCAGTCCAGGTG	2e

Supplementary Table 3.

Spacer sequences at endogenous sites.

**Base editor**	**Site**	**Spacer sequence**	**Figure**
ceRBERESCUE-S	PPIB	GCCAAACACCACATGCTTGCCATCCAGCCAGGCTGTCTTGACTGTCGTGA	3b
RESCUE-S	PPIB	CCACATGCTTGCCATCCAGCCAGGCTGTCTTGACTGTCGTGATGAAGAAC	3b
ceRBERESCUE-S	KRAS-S1	CTTGCCTACGCCACCAGCTCCAACCACCACAAGTTTATATTCAGTCATTT	3b
RESCUE-S	KRAS-S1	CGCCACCAGCTCCAACCACCACAAGTTTATATTCAGTCATTTTCAGCAGG	3b
ceRBERESCUE-S	KRAS-S2	GAGCCTGTTTTGTGTCTACTGTTCCAGAAGGCAAATCACATTTATTTCCT	3b
RESCUE-S	KRAS-S2	TTTGTGTCTACTGTTCCAGAAGGCAAATCACATTTATTTCCTACTAGGAC	3b
ceRBERESCUE-S	SMAD4-S1	CGGCATGGTATGAAGTACTTCGTCCAGGAGCTGGAGGGCCCGGTGTAAGT	3b
RESCUE-S	SMAD4-S1	TATGAAGTACTTCGTCCAGGAGCTGGAGGGCCCGGTGTAAGTGAATTTCA	3b
ceRBERESCUE-S	SMAD4-S2	AATTAGGTGTGTATGGTGCAGTCCCACTTCCAGTCCAGGTGGTAGTGCTG	3b
RESCUE-S	SMAD4-S2	GTGTATGGTGCAGTCCCACTTCCAGTCCAGGTGGTAGTGCTGTTATGATG	3b
ceRBERESCUE-S	FANCC	GGGGGGTTCGGCTGCCGACATCAGCAATTGCTCTGCCACCATCTCAGCCC	3b
RESCUE-S	FANCC	CGGCTGCCGACATCAGCAATTGCTCTGCCACCATCTCAGCCCATCCTCCG	3b
ceRBERESCUE-S	KRAS	ATTCCTCCACAAAATGATTCTGAATTAGCT	3c
ceRBERESCUE-S	NFKB1	TGTGCTTGAAATACTTCTGGATTAAATATT	3c
ceRBERESCUE-S	PPIB	TTGCCTTCGGAGAGGCGCAGCATCCACAGG	3c
ceRBERESCUE-S	SMN1	TTCTCACCAAATGGCAGAACATTTGTCCCC	3c
ceRBERESCUE-S	F8	GCCACACTGATGGACTATTCTCAATTAATA	3c
ceRBERESCUE-S	NF1	TCCACAGGATTCCGGATTGCCATAAATACT	3c
ceRBERESCUE-S	NF2	GAACCTCTTGGGTTGCTTCCTCTTGAGAGA	3c
ceRBERESCUE-S	RAF1	GTAGCAGAGATGCAGCTGGAGCCATCAAAC	3c

ceRBEE488Q/T375S	SMAD4-S2	AATTAGGTGTGTATGGTGCAGTCCcACTTCCAGTCCAGGTGGTAGTGCTG	3d,s2
REPAIR v2	SMAD4-S2	AATTAGGTGTGTATGGTGCAGTCCcACTTCCAGTCCAGGTGGTAGTGCTG	3d,s2

Supplementary Table 4.

sgRNA sequences employed in the *DMD* site.

**sgRNA**	**Sequence**	**Figure**
sgRNA1 for DMDQ1392X mice model	CTTCTTCTATTTAGGCTGTG	s3c
sgRNA2 for DMDQ1392X mice model	AGCTTATATCACTGACAAGG	s3c
**gRNA for DMD Q1392X**	**Spacer Sequence**	**Figure**
mis10	GCTGCCAACcaCTTGTCAATGAATGTGAGGGACTCCTGGATTAAGTGTAA	s3b,4
mis13	TAAGCTGCCAACcaCTTGTCAATGAATGTGAGGGACTCCTGGATTAAGTG	s3b
mis16	ATATAAGCTGCCAACcaCTTGTCAATGAATGTGAGGGACTCCTGGATTAA	s3b
mis19	GCAATATAAGCTGCCAACcaCTTGTCAATGAATGTGAGGGACTCCTGGAT	s3b
mis22	TCTGCAATATAAGCTGCCAACcaCTTGTCAATGAATGTGAGGGACTCCTG	s3b
mis25	TTGTCTGCAATATAAGCTGCCAACcaCTTGTCAATGAATGTGAGGGACTC	s3b
mis28	ACCTTGTCTGCAATATAAGCTGCCAACcaCTTGTCAATGAATGTGAGGGA	s3b
mis31	TCCACCTTGTCTGCAATATAAGCTGCCAACcaCTTGTCAATGAATGTGAG	s3b
mis34	GCGTCCACCTTGTCTGCAATATAAGCTGCCAACcaCTTGTCAATGAATGT	s3b
mis37	GCTGCGTCCACCTTGTCTGCAATATAAGCTGCCAACcaCTTGTCAATGAA	s3b
mis40	TGAGCTGCGTCCACCTTGTCTGCAATATAAGCTGCCAACcaCTTGTCAAT	s3b

Supplementary Table 5.

PCR primers used in the article.

Primer	Sequence	Figure
PPIB-OF	TCAAGGACTTCATGATCCAG	2e,3b
PPIB-OR	TACTCCTTGGCGATGGCAAA	2e,3b
PPIB-IF	GAGACTTCACCAGGGGAGAT	2e,3b
PPIB-IR	TTCTCCACCTCGATCTTGCC	2e,3b
KRAS-S1-OF	TACTCCCGGCCCCCGCCATTT	3b
KRAS-S1-OR	AGAAAGCCCTCCCCAGTCCT	3b
KRAS-S1-IF	TTCGGACTGGGAGCGAGCG	3b
KRAS-S1-IR	CCCTCATTGCACTGTACTCC	3b
KRAS-S2-OF	GGGACCAGTACATGAGGACT	3b
KRAS-S2-OR	TCACACAGCCAGGAGTCTTT	3b
KRAS-S2-IF	GGAGGGCTTTCTTTGTGTAT	3b
KRAS-S2-IR	CAATCTGTATTGTCGGATCT	3b
SMAD4-S1-OF	TGGCAGGAAACATCCCTGGCC	3b
SMAD4-S1-OR	TCTGAGCCATGCCTGACAAGT	3b
SMAD4-S1-IF	AGCTATCAGTCTGTCAGCTG	3b
SMAD4-S1-IR	CCAGTTTCTGTCTGCTAGGA	3b
SMAD4-S2-OF	TCTGTTAGCCCCATCTGAGT	2e,3b
SMAD4-S2-OR	GTCTCTCCTACCTGAACATC	2e,3b
SMAD4-S2-IF	TAATGCTACCAGCACTGCCA	2e,3b
SMAD4-S2-IR	TGGAACACCAATACTCAGGA	2e,3b
FANCC-OF	ACTTCTCCATCTCTTGCCATG	3b
FANCC-OR	TGCTACCGTCTGCAGGTCCTG	3b
FANCC-IF	CTCAAGATATCCCTCGGGGA	3b
FANCC-IR	GCTGCTGCTTCTGGACATTG	3b
KRAS-OF	TACTCCCGGCCCCCGCCATTT	3c
KRAS-OR	AGAAAGCCCTCCCCAGTCCT	3c
KRAS-IF	TTCGGACTGGGAGCGAGCG	3c
KRAS-IR	CCCTCATTGCACTGTACTCC	3c
NFKB1-OF	CCGCCTCTTCCTTCTCCA	3c
NFKB1-OR	CTAGAGGCACCAGGTAGT	3c
NFKB1-IF	CTTAGGAGGGAGAGCCCA	3c
NFKB1-IR	GGTAGTCCACCATGGGATG	3c
PPIB-OF	AGCCCGCGAGCAACCCCA	3c

PPIB-OR	CCGGCCTACATCTTCATCTCC	3c
PPIB-IF	GCGCCGGCTCCCTAGCCA	3c
PPIB-IR	CTTGACGGTGACTTTGGG	3c
SMN1-OF	GCAGGAGGATTCCGTGCT	3c
SMN1-OR	GGAGACCTGGAGTTCTCAC	3c
SMN1-IF	CCAGAGCGATGATTCTGAC	3c
SMN1-IR	GGGAAAGTAGATCGGACAG	3c
F8-OF	GTATCGTCAACAGAGAGTGG	3c
F8-OR	GAGAGTTCTTTCCATGAGTCC	3c
F8-IF	GGACCTGCTTTGTTGACT	3c
F8-IR	CCTTTGTATCCACCTTGCTG	3c
NF1-OF	GCAAGGCACTTGAGAGTTG	3c
NF1-OR	GTAGGCCACGCTCTGTGT	3c
NF1-IF	GGACTCGCCTCTGCACAA	3c
NF1-IR	AGGTGAAGGATGCCTGTACC	3c
NF2-OF	CCACCATGGTGGCCCTGA	3c
NF2-OR	AAGAACCAGGTTTCTCGGAG	3c
NF2-IF	GCCTGTGCAGCAACTCCA	3c
NF2-IR	CTCGGAGCCCCAGAGTCC	3c
RAF1-OF	GGCTCCCTCAGGTTTAAG	3c
RAF1-OR	AGAAGTCTGAACACTGCAC	3c
RAF1-IF	AAGCTGCATCAATGGAGC	3c
RAF1-IR	AGGCAGTCATGCAAGCTC	3c
in vitro DMD-RT-PCR_F	CTGTACAAGGGATCCAGTGG	s3b
in vitro DMD-RT-PCR_R	CCAGATCTTTGAGCTGCGTCCAC	s3b
in vivo DMD-RT-PCR_F	AATCAGATTCGTCTATTGGCACA	4b
in vivo DMD-RT-PCR_R	CCCTTTGGTTGGCATCCTT	4b
Genotyping of DMDQ1392X mice_F	ATTCATATAGGGCTTCAGTTCC	s3c
Genotyping of DMDQ1392X mice_R	CATCTGTTTTAATAGTGTGCAT	s3c
